# Supplementary figures and images for: A role for reward valuation in the serotonergic modulation of impulsivity
Source: Psychopharmacology (Berl). Author manuscript; Available in PMC 2022 Jan 6. (PMC8605981; doi:10.1007/s00213-021-05944-2)

**
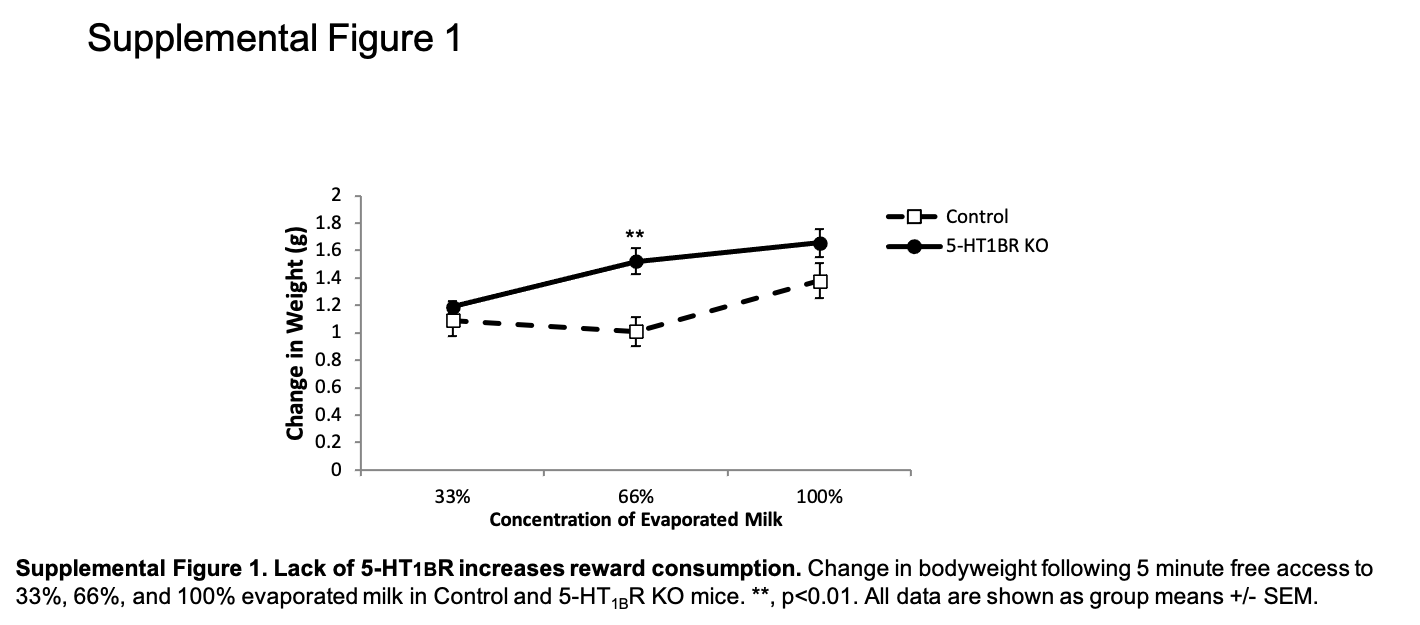
**

**
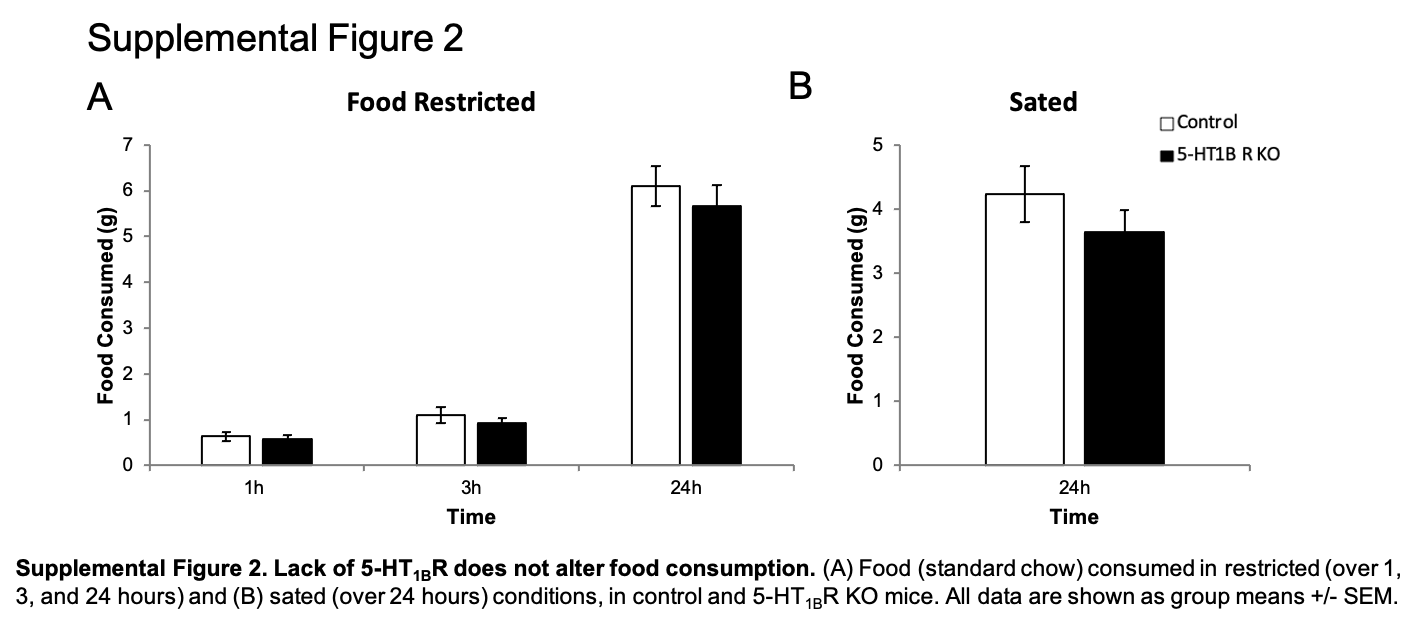
**

**
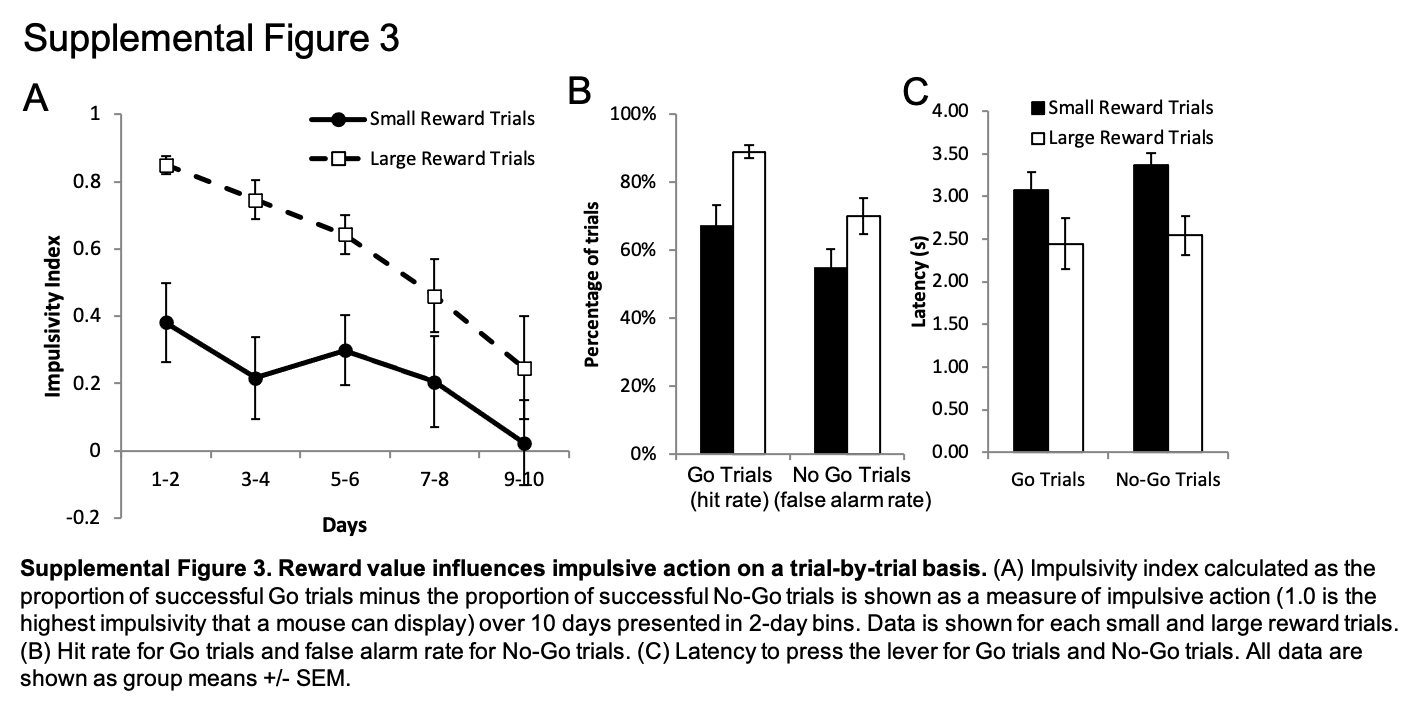
**


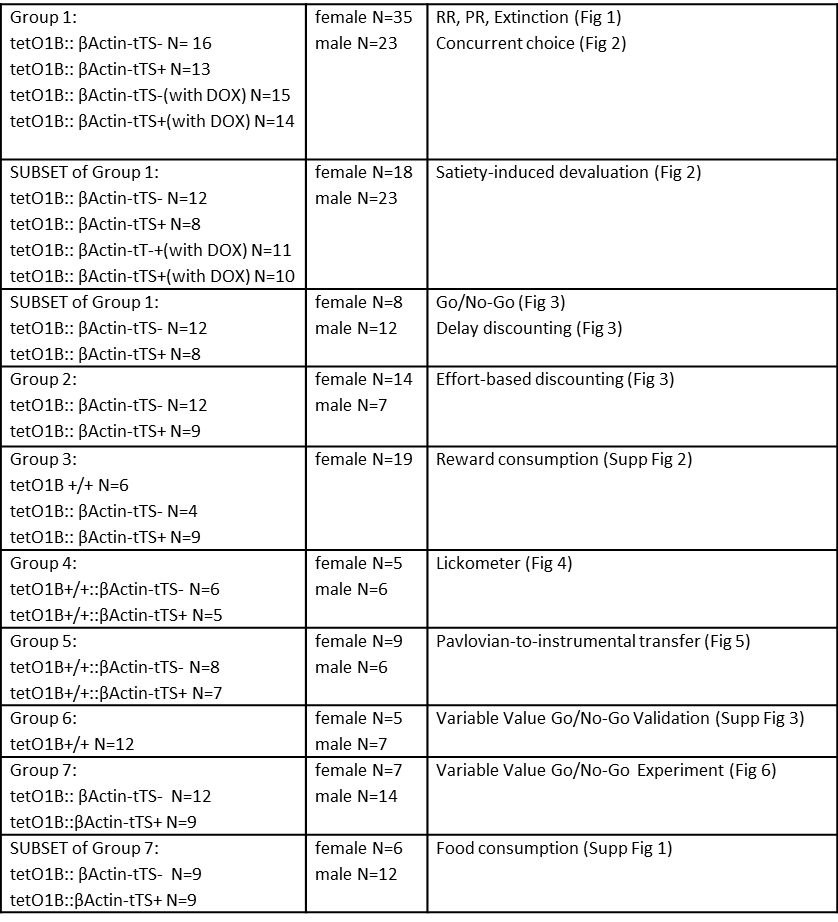


Supplemental Table 1

Supplement: Supplementary Material [file NIHMS1760773-supplement-Supplementary_Material.docx]
